# The Ecology of Medical Care in Beijing

**DOI:** 10.1371/journal.pone.0082446

**Published:** 2013-12-05

**Authors:** Shuang Shao, FeiFei Zhao, Jing Wang, Lei Feng, XiaoQin Lu, Juan Du, YuXiang Yan, Chao Wang, YingHong Fu, JingJing Wu, XinWei Yu, KayKeng Khoo, YouXin Wang, Wei Wang

**Affiliations:** 1 General Practice and Continue Education School, Capital Medical University, Beijing, China; 2 Beijing Municipal Key Laboratory of Clinical Epidemiology, School of Public Health, Capital Medical University, Beijing, China; 3 School of Yan Jing Medical Sciences, Capital Medical University, Beijing, China; 4 Laboratory of Parallel Software and Mathematic Science, Institute of Software, Chinese Academy of Sciences, Beijing, China; 5 Beijing Nuclear Industry Hospital, Beijing, China; 6 Lynwood Medical Centre, Lynwood, Western Australia, Australia; 7 School of Medical Sciences, Edith Cowan University, Perth, Australia; Iran University of Medical Sciences, Islamic Republic of Iran

## Abstract

**Background:**

We presented the pattern of health care consumption, and the utilization of available resources by describing the ecology of medical care in Beijing on a monthly basis and by describing the socio-demographic characteristics associated with receipt care in different settings.

**Methods:**

A cohort of 6,592 adults, 15 years of age and older were sampled to estimate the number of urban-resident adults per 1,000 who visited a medical facility at least once in a month, by the method of three-stage stratified and cluster random sampling. Separate logistic regression analyses assessed the association between those receiving care in different types of setting and their socio-demographic characteristics.

**Results:**

On average per 1,000 adults, 295 had at least one symptom, 217 considered seeking medical care, 173 consulted a physician, 129 visited western medical practitioners, 127 visited a hospital-based outpatient clinic, 78 visited traditional Chinese medical practitioners, 43 visited a primary care physician, 35 received care in an emergency department, 15 were hospitalized. Health care seeking behaviors varied with socio-demographic characteristics, such as gender, age, ethnicity, resident census register, marital status, education, income, and health insurance status. In term of primary care, the gate-keeping and referral roles of Community Health Centers have not yet been fully established in Beijing.

**Conclusions:**

This study represents a first attempt to map the medical care ecology of Beijing urban population and provides timely baseline information for health care reform in China.

## Introduction

The theory of “the ecology of medical care” was first proposed by White in 1961, providing a framework of organizing complex factors known to affect health care. This conceptual framework offered a useful tool to health care policy makers and direction for future research [1,2]. Understanding ecological relationships can also guide assessments of the adequacy, effectiveness, efficiency and appropriateness of the existing health care resources [3,4].

In the last few decades, several reports assessed the progress of China’s healthcare delivery systems, in the wake of rapid economic growth, poverty reduction, significantly increased life expectancy, massively decreased infant mortality rate, and burgeoning healthcare coverage [5]. However these reports also indicated that China’s health outcome has exhibited several undesirable features: such as growing inequalities in access to health care and different health status across regions of different socioeconomic standings and between urban and rural areas. Hospital commercialization and deficiencies in the supply of medical care have driven up the cost of medical care delivery, thus further increased the barrier to access of medical care [6-8].

Facing a series of deeply rooted problems, Chinese government undertook phased measures in 2009 to achieve universal health care coverage by 2020 [9]. In the first phase of 2009 to 2011, the reform is anchored in five interdependent areas: expanding coverage to insure more than 90% of the population, establishing a national essential medicines system to meet everyone’s primary needs of medicine, improving the primary care delivery system to provide basic health care and to manage referrals to specialist care and hospitals, making public health services available and equal for all, and piloting public hospital reforms [10,11]. The data of National Health Services Survey (NHSS) reported that an impressive expansion of medical insurance coverage has increased from 29.7% in 2003 to 95.7% in 2011, covering about 1.28 billion people, and narrowing the inequality-gaps in accessing health services between rural and urban areas. However, increased insurance coverage has not yet been effective in reducing patients’ financial risks, as both health expenditure and out-of-pocket payments continue to rise rapidly. In 2011, the inpatient reimbursement level was relatively modest, about 50%, and 13% of households reported catastrophic health expenditures, showing health expenses as a percentage of total household expenditure continue to increase after the 2009 health reform [12]. During the first period, most of the researches drew attention to developments made in several areas of health system reform (HSR), but none of the reports attempted to predict progress towards achieving the major HSR objective, which is equitable and affordable access to quality health service.

In this study, we applied the quantitative socio-epidemiology method to quantify the variation among the subsets of Beijing population seeking medical care in one month and analyzed the influences of illness on the care seeking behavior of the population. 

## Materials and Methods

### Ethics Statement

The study was approved by the Ethical Committee of Capital Medical University, Beijing, China. Written informed consent was obtained from each participant involved in this study. For participants under the age of eighteen, written informed consents were obtained from their guardians. All participants' information was kept confidential and tracked anonymously with identification number only.

### Data acquisition and study population

Data for the Beijing urban residents were taken from a cross-sectional survey of 2012-Beijing Urban Population Health Care Service. The study was undertaken as a part of the National High Technology Research and Development Program-863 of the Chinese Government, which is a population-based cross-sectional survey on risk factors of chronic non-communicable diseases (NCD). 

The survey studied urban-residents of Beijing, who were 15 years old and over, including both urban residents with Beijing resident certificate “*Hukou*” and inter-provincial migrants, but excluding visitors to Beijing, and those mentally unfit to respond to the survey. Those under 15 years of age were not included in this study because decisions about their medical care are customarily made by their parents or guardians. Fieldwork was carried out during a period of three months from March to May, 2012. Quality assurance measures for the survey included proper training of enumerators, and monitoring of the interviewing process by fieldwork supervisors. The completed questionnaires, and validation of the collected data were independently checked. A pilot survey covering 400 persons was conducted between February 26 to 29, 2012 to assess applicability of the questionnaire and the fieldwork procedures.

**Figure 1:**
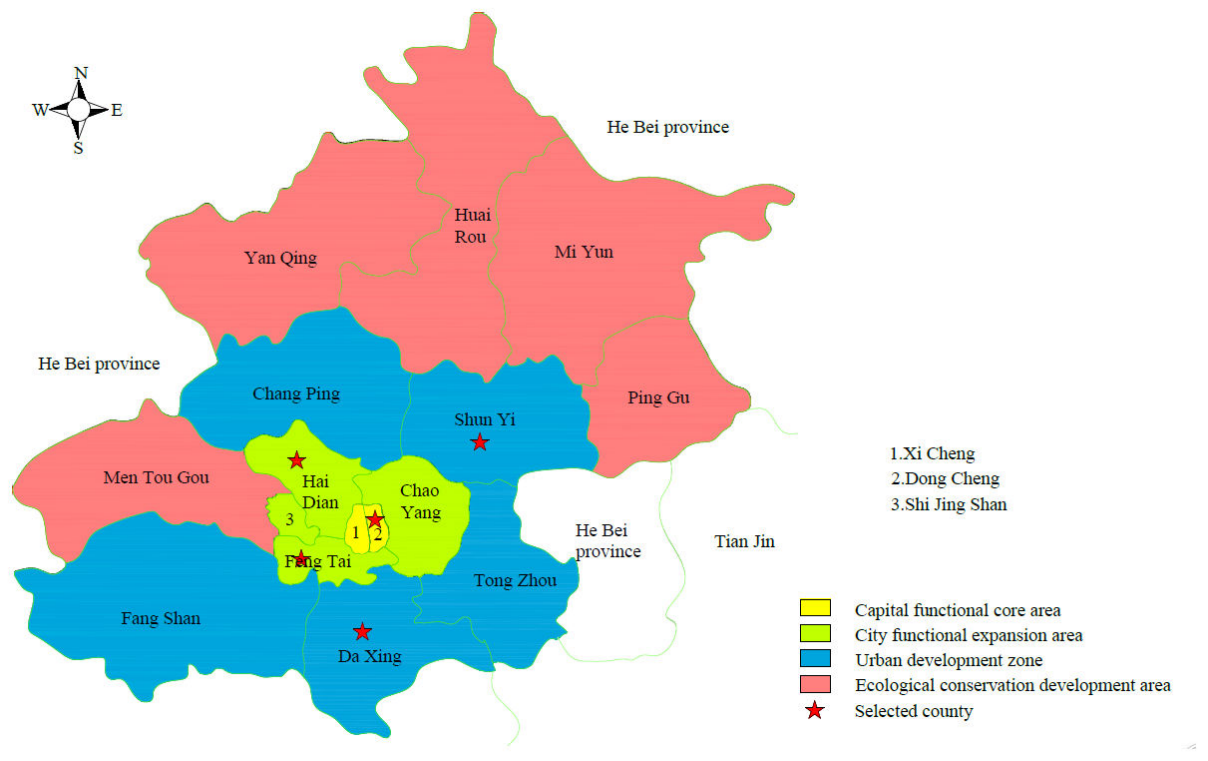
The map of Beijing.

A population-weighted sample of 7,000 adults was chosen from 5 out of 16 counties in Beijing by the method of three-stage stratified and cluster random sampling. Beijing urban residents dwell mainly in three out of four areas classified according to the functions of the county namely capital function core area, city functional expansion area, urban development zone and ecological conservation development area. In the first stage, one county was drawn from the capital functional core area (*Dongcheng* district), two counties were drawn from the city functional expansion area (*Haidian* and *Fengtai* districts), and another two counties were drawn from the urban development zone (*Daxing* and *Shunyi* districts) (Figure 1). In the second stage, two streets according to the economic condition and population size from each selected county were selected. Finally, for each street selected, the total number of adults extracted and investigated was 7,000. The population sampled in *Dongcheng, Haidian, Fengtai, Daxing* and *Shunyi* was 1000, 2000, 2000, 1000 and 1000 respectively. All adults were approached for face-to-face interview. We excluded those adults whose background information was missing on any of the study variables. Finally, data of 6,592 of the 7,000 respondents were included in this study. 

### Statistical analysis

**Figure 2:**
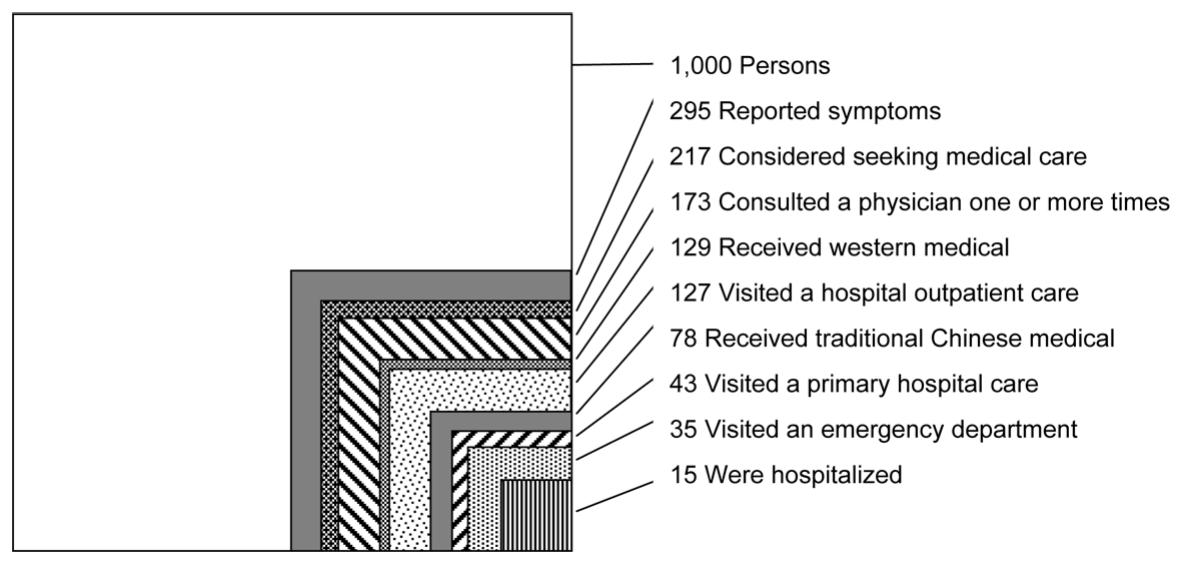
Monthly prevalence estimates of illness and the roles of different settings in the health-care utilization. Data are for the Beijing urban resident population in 2012 (adult 15 years of age and older). Each box does not necessarily represent a subgroup of the larger box, i.e., some values are overlapping. The values are based on 1,000 persons.

Descriptive analyses were performed using the ecology model [2], to estimate the number of individuals per 1,000 of the Beijing urban residents who had experienced the different health care seeking events as shown in Figure 2. We also described the differences of health care seeking behaviors between residents with Beijing “*Hukou*” and inter-provincial migrants respectively in table 1. Definitions of terms are summarized in the Appendix (See table S1).

**Table 1:** The symptoms and levels of distress or incapacitation experienced for persons who experienced illness.

	**Number**	**Percent (%)**
**Symptom**		
Fever	312	16.5
Pain	589	30.3
Diarrhea	113	5.8
Cough	564	29.0
Cardiopalmus	321	16.5
Other	437	22.5
**Level**		
Not serious	598	30.8
General	1090	56.1
Serious	256	13.1

A person-month was the unit of analysis and indicated receipt of services in a health care setting at least once in a month, but not the total number of times care was received in the setting during the month. For each survey participant, the individual’s survey weight was totaled for each record in which one type of event occurred, and the result was multiplied by 1,000 to produce the estimates. Separate logistic regressions were used to derive the adjusted odds ratios of the respective predictor variables on the receipt of care in each setting. We assessed the association of every variable of receiving care in each of the seven health care settings while statistically controlling all other predictor variables. For each variable, the most populated category was designated as the reference group in the development of adjusted odds ratios. 

All analyses were conducted using the Statistical Package for Social Science (SPSS) for Windows (Version 17.0; SPSS Inc., Chicago, IL, USA) and all the tests are two sided. *P*<0.05 was considered statistically significant.

## Results

A total of 6,592 adults (male 2,373 and female 4,219, 15 years and older) of Beijing urban residents were enumerated and interviewed comprising 4,578 residents (males 1,654; females 2,924) with Beijing “*Hukou*” and 2,014 inter-provincial migrants (males 719; females 1,295). Applying the population weights for both subgroups, respectively, the total sample represented 13,942,393 persons of the local urban population resident in Beijing in 2010 [13].

Figure 2 shows the incidence of different health care seeking behaviors in the model of ecology of medical care. We estimated that of 1,000 adults in Beijing per month, 295 experienced at least one episode of discomfort, illness or injury, 217 considered seeking medical cares, 173 consulted a physician one or more times. Compared to 129 adults who visited western medical practitioners, only 78 adults visited traditional Chinese medical practitioners. 127 visited a hospital-based outpatient clinic, while only 43 visited a primary care physician. 35 received care in an emergency department of a hospital, while 15 were hospitalized.

**Figure 3:**
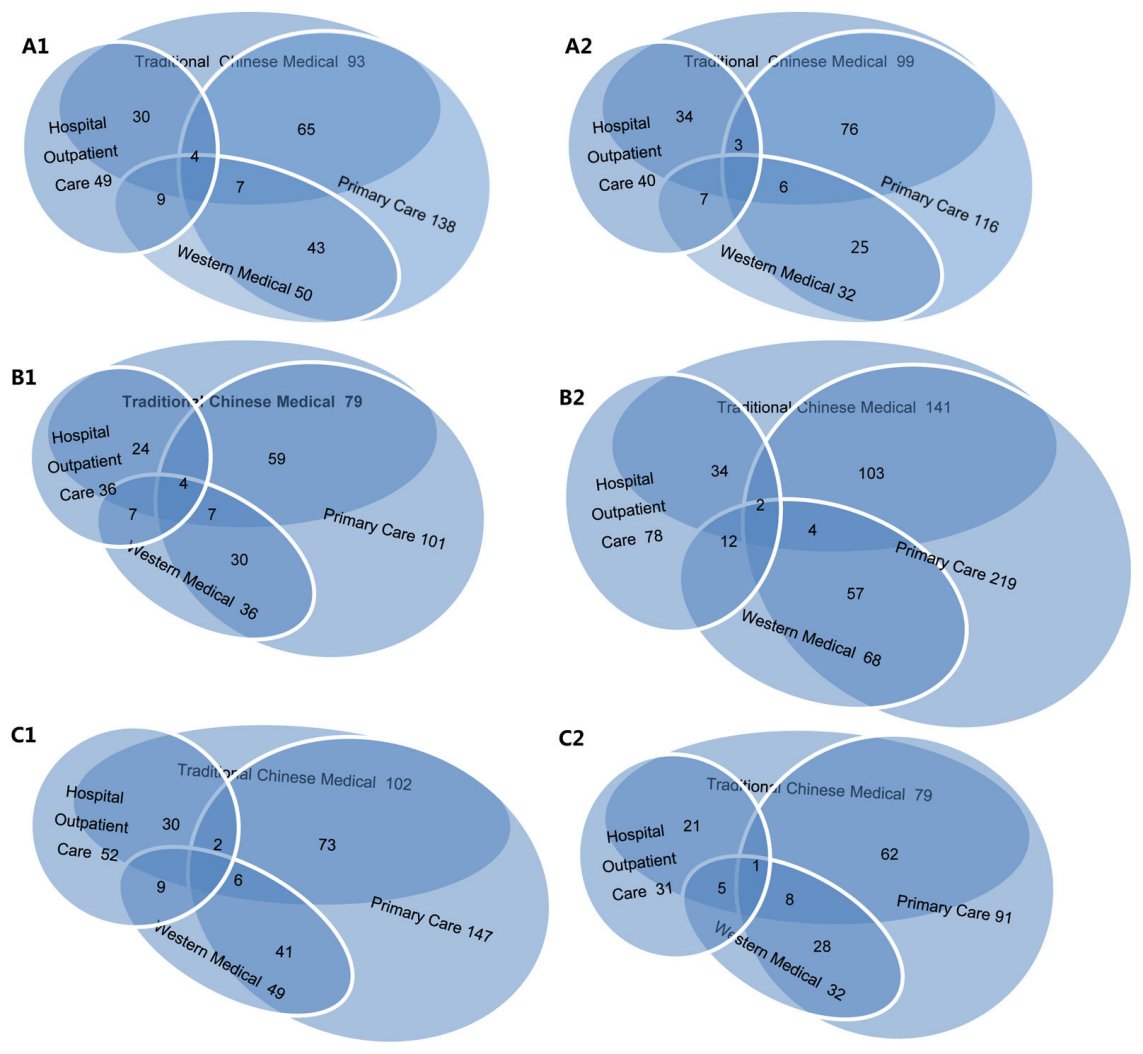
The proportion of subjects who did not experience health problems in four health-care settings. Four health care settings include traditional Chinese medical care, western medical care, primary care and hospital-based outpatient care. A1 is female, A2 is male, B1 is age under 55 years, B2 is age above 55 years, C1 is resident with “Hukou”, C2 is migrant. The values are based on 1,000 persons.

In A1 and A2 of Figure 3, we estimated that of 1,000 females and 1,000 males in Beijing per month, the numbers of those who did not receive traditional Chinese medical care, did not receive western medical care, did not visit a hospital outpatient clinic, and did not visit a primary care were 93, 50, 49, 138 for females, and 99, 32, 40, 116 for males respectively. 65 females and 76 males did not receive neither primary care, nor traditional Chinese medical care; 43 females and 25 males did not receive neither primary care nor western medical care; 30 females and 34 males did not receive neither traditional Chinese medical care nor hospital outpatient care; 9 females and 7 males did not receive neither hospital outpatient care nor western medical care; 7 females and 6 males did not receive neither traditional Chinese medical care nor western medical care; 4 females and 3 males did not receive neither primary care nor hospital outpatient care. 

In the B1 and B2 of Figure 3, we estimated that of 1,000 persons aged under 55 years and 1,000 persons aged above 55 years in Beijing per month, the numbers of those who did not receive traditional Chinese medical care, did not receive western medical care, did not visit a hospital outpatient clinic, and did not visit a primary care were 79, 36, 36, 101 for aged under 55 and 141, 68, 78, 219 for aged over 55 respectively. 59 aged under 55 and 103 aged over 55 did not receive both primary care and traditional Chinese medical care; 30 aged under 55 and 57 aged over 55 did not receive neither primary care nor western medical care; 24 aged under 55 and 34 aged over 55 did not receive neither traditional Chinese medical care nor hospital outpatient care; 7 aged under 55 and 12 aged over 55 did not receive neither hospital outpatient care nor western medical care; 7 aged under 55 and 4 aged over 55 did not receive neither traditional Chinese medical care nor western medical care; 4 aged under 55 and 2 aged over 55 did not receive neither primary care nor hospital outpatient care. 

In the C1 and C2 of Figure 3, we estimated that of 1,000 residents with “*Hukou*” and 1,000 migrants in Beijing per month, the numbers of those who did not receive traditional Chinese medical care, did not receive western medical care, did not visit a hospital outpatient clinic and did not visit a primary care were 102, 49, 52, 147 for residents with “*Hukou*” and 79, 32, 31, 91 for migrants respectively. 73 residents with “*Hukou*” and 62 migrants did not receive both primary care and traditional Chinese medical care; 41 residents with “*Hukou*” and 28 migrants did not receive neither primary care nor western medical care; 30 residents with “*Hukou*” and 21 migrants did not receive neither traditional Chinese medical care nor hospital outpatient care; 9 residents with “*Hukou*” and 5 migrants did not receive neither hospital outpatient care nor western medical care; 6 residents with “*Hukou*” and 8 migrants did not receive neither traditional Chinese medical care nor western medical care; 2 residents with “*Hukou*” and 1 migrants did not receive neither primary care nor hospital outpatient care. 

Table 1 indicates that of 1,944 persons (29.5% of a total of 6,592 persons), 312 (16.5%) experienced fever, 589 (30.3%) experienced pain, 113 (5.8%) experienced diarrhea, 564 (29.0%) experienced cough, 321 (16.5%) experienced cardiopalmus, and 437 (22.5%) experienced other symptoms. In summary, the proportion of serious symptoms was 13.1% (256) and the proportion of general and not serious symptoms were 56.1% (1090) and 30.8% (598) respectively. 

Of 1,944 persons who considered seeking health care, 1,430 persons did so, among whom 1,140 consulted a physician one or more time, 892 purchased drug from hospital, and others received health care services such as rehabilitation, or counseling. 

Table 2 shows that 991 persons (49.2%) of total migrants had no insurance coverage, compared with 412 persons (9.0%) of total residents with Beijing “*Hukou*”. Of 1,000 migrants 250 persons compared with 315 residents with Beijing “*Hukou*” reported having symptoms and they were less likely to take regular medication in all settings. The ratio of health care seeking behavior among migrants was lower (236/504=46.8%) than resident with Beijing “*Hukou*” (902/1,442=62.6%). 

**Table 2:** The insurance coverage and health seeking behaviors of migrants and residents with “*Hukou*” in Beijing.

	**Number (%)**	
	**Migrant**	**Resident with “*Hukou*”**
Insurance coverage		
uninsured	991(49.2)	412 (9.0)
insured	1023 (50.8)	4166 (91.0)
Have one or more symptoms	504 (25.0)	1442 (31.5)
Considered seeking medical care	334(16.6)	1094 (23.9)
consulted a physician	236 (11.7)	902 (19.7)
Traditional Chinese medical visit	79 (3.9)	435 (9.5)
Western medical visit	173 (8.6)	678 (14.8)
Primary care visit	52 (2.6)	229 (5.0)
Hospital outpatient clinic visit	171 (8.5)	664 (14.5)
Hospitalization	12 (0.6)	82 (1.8)
Emergency departments visit	50 (2.5)	183 (4.0)

Table 3 provides the patterns of health care seeking behavior according to gender, age, ethnicity, census register, marital status, education, income, and insurance status in a typical month. When compared with residents with Beijing “*Hukou*”, migrants have a lower rate of utilization of health care in all settings. 

**Table 3:** Variation in the number of persons who received care in different health care settings in Beijing in 2012 (persons per 1,000) by socio-demographic characteristics.

	**Number per 1000 (95% CI)**						
**Characteristics**	**Have one or more symptoms**	**Traditional Chinese medical visit**	**Western medical visit**	**Primary care visit**	**Hospital outpatient clinic visit**	**Hospitalization**	**Emergency departments visit**
**Sex**							
Male	280(262-298)	55(46-64)	122(109-135)	38(30-46)	113(100-126)	18(13-23)	37(29-45)
Female	303(289-317)	91(82-100)	133(123-143)	46(40-52)	134(124-144)	13(10-16)	35(29-41)
**Age**							
15-24y	248(236-260)	29(8-50)	88(77-99)	31(12-50)	76(64-88)	9(3-15)	35(29-41)
25-34y	235(215-255)	41(32-50)	86(73-99)	30(22-38)	77(64-90)	5(2-8)	23(16-30)
35-44y	270(246-294)	44(33-55)	93(77-109)	36(26-46)	96(80-112)	11(5-17)	30(21-39)
45-54y	298(272-324)	97(80-114)	122(103-141)	29(19-39)	143(123-163)	16(9-23)	26(17-35)
55-64y	374(347-401)	147(128-166)	208(186-230)	75(61-89)	195(173-217)	27(18-36)	52(40-64)
≥65y	482(430-534)	175(136-214)	294(247-341)	80(52-108)	294(247-341)	39(19-59)	83(55-111)
**Ethnicity**							
Han	291(280-302)	76(69-83)	129(121-137)	42(37-47)	127(119-135)	15(12-18)	34(30-38)
Minority	364(313-415)	108(75-141)	125(90-160)	61(36-86)	117(83-151)	12(5-24)	52(29-75)
**Census register**							
Residents with Beijing resident certificate (*Hukou* )	315(302-328)	95(87-103)	148(138-158)	50(44-56)	145(135-219)	18(14-22)	40(34-46)
Migrants	250(231-269)	39(31-47)	85(73-97)	25(26-94)	85(73-97)	6(3-9)	25(18-32)
**Marital status**							
Unmarried	251(228-274)	42(32-52)	91(76-106)	30(21-39)	83(69-97)	8(3-13)	28(19-37)
Married	299(286-312)	85(77-93)	133(123-143)	43(37-49)	133(123-143)	15(12-18)	36(31-42)
Divorced	356(274-438)	114(60-168)	182(116-248)	61(20-102)	189(122-256)	15(5-35)	45(10-80)
Widowed	448(381-515)	138(91-185)	252(193-311)	100(59-141)	243(185-301)	43(156-70)	52(22-82)
**Education**							
Master’s degree or above	315(273-357)	58(37-79)	120(90-150)	30(15-45)	122(92-152)	13(3-23)	36(19-53)
University or college	292(274-310)	70(60-80)	122(109-135)	35(28-42)	121(108-134)	9(5-13)	31(24-38)
High school or secondary	269(251-287)	73(62-84)	113(84-100)	43(35-51)	114(101-127)	14(9-19)	31(24-38)
Junior high school	304(278-330)	99(82-116)	153(133-173)	55(42-68)	142(122-162)	24(15-33)	39(28-50)
Primary school and below	470(408-531)	127(86-168)	239(186-292)	80(46-114)	239(186-292)	36(13-59)	96(60-132)
**Incoming**							
<1000	308(276-340)	55(39-71)	124(101-147)	38(25-51)	120(97-143)	25(14-36)	39(25-53)
1000-2999	310(294-326)	97(86-108)	147(134-160)	50(42-58)	145(132-158)	16(12-20)	38(31-45)
3000-4999	286(265-307)	71(59-83)	118(103-133)	35(27-43)	117(102-132)	9(5-13)	30(22-38)
5000-9999	243(213-273)	50(35-65)	96(75-117)	46(31-61)	76(57-95)	8(2-14)	30(18-42)
>10000	295(235-355)	50(21-79)	105(64-146)	9(3-15)	150(103-197)	27(6-48)	41(15-67)
**Insurance status**							
Insured	305(292-318)	88(80-96)	139(130-148)	46(40-52)	136(127-145)	15(12-18)	36(31-41)
Uninsured	257(234-280)	41(31-51)	93(78-109)	29(20-38)	91(76-106)	13(7-19)	33(24-42)

Abbreviation: CI: confidence interval.Note: Categories of monthly incoming based on Chinese incoming criteria and Chinese currency RMB unite Yuan ($1=~RMB 6.30).

Table 4 displays the adjusted odds ratios (ORs) of receiving care in each setting in a typical month, isolated to each variable while controlling for all other prediction variables. Selected findings were organized by the predictor variables, first noting variation and then odds ratios. Female was also an independent predictor of receiving services in traditional Chinese medicine (TCM) visits (OR = 1.75, 95% CI 1.35, 2.26, *P*<0.001) and hospitalization (OR=0.58, 95% CI 0.38, 0.88, *P*<0.05). Age was a strong independent predictor of receiving health care in TCM visits. Migration status was an independent predictor of better self-reported health (OR=0.73, 95% CI 0.64, 0.82, *P*<0.001), and lower likelihood of receiving health care in TCM visits (OR=0.54, 95% CI 0.40, 0.72, *P*<0.001). Being married did predict a higher likelihood of receiving care in TCM visits. Insured population reported significantly more than uninsured seeking TCM therapy (*P*<0.001). 

**Table 4:** Adjust OR that a person with particular characteristics received medical care in selected settings in Beijing in a typical month in 2012.

	**Adjust OR (95% CI)**						
**Variables**	**Have one or more symptoms**	**Traditional Chinese medical visit**	**Western medical visit**	**Primary care visit**	**Hospital outpatient clinic visit**	**Hospitalization**	**Emergency departments visit**
**Sex**							
Male	1.0	1.0	1.0	1.0	1.0	1.0	1.0
Female	1.12(1.00-1.25)	1.75(1.35-2.26)*	0.68(0.50-0.92)	1.03(0.77-1.37)	1.03(0.77-1.36)	0.58(0.38-0.88)†	0.75(0.55-1.01)
**Age**							
15-24y	1.0	1.0	1.0	1.0	1.0	1.0	1.0
25-34y	0.93(0.77-1.13)	1.69(0.98-2.91)	1.02(0.56-1.84)	1.03(0.59-1.78)	1.10(0.65-1.87)	0.49(0.18-1.35)	0.59(0.34-1.03)
35-44y	1.12(0.92-1.37)	1.37(0.79-2.39)	0.61(0.34-1.09)	0.98(0.56-1.73)	1.13(0.66-1.94)	0.98(0.39-2.42)	0.63(0.36-1.10)
45-54y	1.29(1.06-1.58)†	3.50(2.05-5.96)*	0.64(0.36-1.12)	0.54(0.30-0.97)†	2.07(1.19-3.61)†	1.06(0.45-2.54)	0.38(0.22-0.69)‡
55-64y	1.82(1.50-2.20) *	3.36(2.03-5.55)*	0.91(0.53-1.56)	1.02(0.61-1.69)	1.21(0.74-1.97)	1.19(0.53-2.66)	0.52(0.31-0.86)†
≥65y	2.83(2.18-3.66)*	2.49(1.41-4.38)‡	1.02(0.53-1.93)	0.74(0.40-1.36)	1.74(0.96-3.15)	1.26(0.51-3.13)	0.63(0.35-1.14)
**Ethnicity**							
Han	1.0	1.0	1.0	1.0	1.0	1.0	1.0
other	1.40(1.11-1.75)‡	1.87(1.01-3.14)†	0.75(0.43-1.32)	1.61(0.93-2.77)	0.64(0.38-1.10)	0.75(0.27-2.10)	1.64(0.93-2.89)
**Census register**							
Resident with Beijing resident certificate (*Hukou* )	1.0	1.0	1.0	1.0	1.0	1.0	1.0
Migrant	0.73(0.64-0.82)*	0.54(0.40-0.72)*	0.88(0.64-1.22)	0.81(0.58-1.14)	0.99(0.72-1.37)	0.58(0.32-1.06)	0.94(0.66-1.34)
**Marital status**							
Unmarried	1.0	1.0	1.0	1.0	1.0	1.0	1.0
Married	1.28(1.11-1.46)*	1.63(1.15-2.30)‡	0.83(0.56-1.23)	0.94(0.64-1.38)	1.20(0.83-1.72)	1.32(0.68-2.54)	0.80(0.54-1.19)
Divorced	1.65(1.13-2.40)‡	1.51(0.71-3.22)	0.77(0.33-1.79)	0.95(0.40-2.25)	1.31(0.55-3.10)	0.90(0.19-4.28)	0.70(0.27-1.82)
Widowed	2.42(1.80-3.25)*	1.22(0.69-2.16)	0.80(0.42-1.52)	1.22(0.66-2.25)	1.017(0.55-1.87)	2.00(0.79-5.06)	0.57(0.27-1.18)
**Education**							
Master’s degree or above	0.52(0.38-0.71)*	0.86(0.50-1.65)	1.04(0.50-2.16)	0.70(0.32-1.51)	1.12(0.53-2.35)	0.70(0.24-2.06)	0.70(0.39-1.43)
University or college	0.47(0.36-0.61)*	1.18(0.72-1.93)	1.09(0.62-1.90)	0.84(0.48-1.47)	1.03(0.59-1.79)	0.46(0.20-1.03)	0.56(0.33-0.96)
High school or secondary	0.42(0.32-0.54)*	1.30(0.80-2.14)	0.85(0.49-1.47)	1.12(0.64-1.95)	0.86(0.49-1.49)	0.78(0.36-1.71)	0.58(0.34-1.00)
Junior high school	0.49(0.37-0.65)*	1.44(0.86-2.41)	1.04(0.58-1.87)	1.12(0.63-2.00)	0.79(0.44-1.41)	1.05(0.47-2.34)	0.56(0.31-0.99)
Primary school and below	1.0	1.0	1.0	1.0	1.0	1.0	1.0
**Incoming**							
<1000	1.0	1.0	1.0	1.0	1.0	1.0	1.0
1000-2999	1.01(0.85-1.19)	1.83(1.22-2.73)‡	0.83(0.53-1.32)	1.07(0.68-1.67)	0.91(0.58-1.41)	0.46(0.26-0.80)‡	0.56(0.31-0.99)
3000-4999	0.90(0.75-1.08)	1.60(1.03-2.47)†	0.89(0.54-1.46)	0.92(0.56-1.50)	0.99(0.61-1.62)	0.31(0.16-0.63)‡	0.58(0.34-1.00)
5000-9999	0.72(0.58-0.90)‡	1.23(0.71-2.13)	0.88(0.47-1.64)	1.79(1.00-3.20)	0.49(0.28-0.87)†	0.35(0.13-0.90)†	0.56(0.33-0.96)
>10000	0.94(0.68-1.31)	0.84(0.38-1.87)	0.51(0.23-1.14)	0.19(0.04-0.82)†	3.63(1.04-12.66)†	1.08(0.40-2.93)	0.70(0.34-1.43)
**Insurance status**							
Insured	1.0	1.0	1.0	1.0	1.0	1.0	1.0
Uninsured	0.79(0.69-0.90)‡	0.55(0.39-0.77)*	0.95(0.66-1.37)	0.91(0.63-1.33)	0.93(0.65-1.33)	1.28(0.75-2.20)	1.46(1.01-2.13)†

Abbreviation: CI: confidence interval.Note: *P <0.001, ‡*P* <0.01, †*P* <0.05.

## Discussion

This study represents a first attempt to map the medical care ecology of Beijing urban population. Data on ‘‘natural history’’ of health care describe the patterns of illness, distribution of illness episodes in relation to self-care, care-seeking behavior and health care consumption at different levels of the health-care delivery system. Although our researches do not describe the total number of health seeking behaviors monthly, which may reduce the actual utilization, we focus on the trend of first time health seeking behaviors in all settings.

The results derived from our study reflect the overall patterns of health care seeking behaviors in Beijing in 2012. The monthly estimates for various types of health events in Beijing (fig.2) show substantial differences to the report of US findings in 2001. There were fewer people in the community reporting symptoms in one month in Beijing (295 per 1,000) compared to the US (800 per 1,000). Such a big difference may be partly due to the large submerged part of the clinical iceberg [14], for example, the prevalence of chronic kidney disease in China has been estimated to be 119.5 million, but only 12.5% of these individuals are aware of their condition [15]. Another reason for under-reporting may be due to the phenomenon of “suboptimal health status” (SHS) [16,17], which is a health status without a proper diagnosis, but is actually associated with a major chronic disease.

Labor migration has played an important role in the rapid economic growth and urbanization process in China in the last few decades. The figure of 7.05 million migrants accounting for up to a third of Beijing population in 2010 is increasing at an annual rate of 3.8% in the last ten years. Large-scale population movements present stress for health systems and public health in terms of disease monitoring, prevention and treatment [18]. Basic medical services in China have been recognized as one of the “essential rights of the people”. Our data indicate that there were 315 residents with Beijing “*Hukou*” per 1,000 adults compared with 250 migrants per 1,000 adults who reported having symptoms. There might be two factors which may account for this difference. Firstly, migrants who felt greater imperative to work, might be more likely to underreport ill health than their urban counterparts [19]. Secondly, it has been reported that migrants are generally healthier than local population according to the “healthy migrant” theory, which attributes healthier individuals being more likely to relocate to other provinces [20,21]. It is not surprising that younger migrants who are labor migrants in particular tend to be more positively selected with respect to health than the elderly migrants. Furthermore, migrants had better self-rated health, and were less likely to take regular medication. Lower healthcare insurance coverage in Beijing and high health cost increased the barriers to access health care for migrants. Although many migrants had insurance in their home town, it is nearly impossible to transfer insurance relations between different countries in China. 

As one of the oldest health systems, TCM is institutionalized into Chinese health care system and is widely accessible alongside western medicine. Ironically, while interest in herbal remedies and acupuncture has been rapidly increasing in the west in the last few decades, the Chinese public is turning increasingly to western medicine [22]. The increasing interest in western medicine slackened the development of TCM in the early 20^th^ century in China. It has been suggested that unpalatable taste and unattractive colors of herbal medicine reduced the popularity of TCM, while the paucity of randomized controlled trials in TCM rendered it in less favorable light when compared with the increasingly evidence based western medicine [23-26]. In China, western medicineis being utilized more in the treatment of acute conditions such as infectious diseases, while TCM is utilized mainly in the prevention and treatment of non-communicable chronic diseases (NCD) [23,24]. Although the majority of Chinese still seek to understand their illness by way of traditional medical concepts rather than western medical principles, our research suggests that the community’s care-seeking orientation still favors western medicine (129/1,000) over TCM (73/1,000), especially in emergency illness of industrialization and urbanization [27,28]. Our research also indicated that compared with younger persons, the elderly preferred to select TCM. Younger persons preferred western medicine as being more cost-effective with faster and more predictable actions, leading to a favorable resolution in a short period of time.

The perception that “obtaining medical care is both expensive and difficult” was revealed on public opinion polls [29], and the Chinese government responded with setting up equitable, affordable and easily accessible primary health care system by establishing community health centers (CHCs), with the intended objective of directing urban patients’ reliance on hospital services towards primary care services and ensuring universal access to basic primary and public health services. Our data indicated that patients in Beijing under-utilized the services of CHCs (only 43 did so out of 217 who considered seeking medical care) suggesting there is widespread dissatisfaction with the provision of services in CHCs and with the quality of medical services being provided, and this is in agreement with previous published results [8,30]. In order to uplift the quality of General Practice in CHCs, the basic medical training of General Practitioners has to be raised and the scope of training has to be widened and designed to suit the important role of a Primary Care Provider with gate-keeping functions. This will mean additional period of post-graduate supervised mentorship. To encourage urban patients towards utilizing primary health care services, a two-way referral procedure, between secondary/tertiary hospitals and CHCs, was proposed by the State Council in 2006. As one of the first pilot cities, Beijing health authorities realized a well-functioning referral system can decrease cost, increase utilization of CHCs and enhance equity, while the lack of administrative supervision can restrict the development of two-way referral system [31]. Driven by profit earning, hospitals have no motivation to actively transfer patients back to CHCs facilities for follow-up treatment. In term of primary care, the gate-keeping and referral roles have not yet been fully established. 

Economic modernization, state policies and market transition are changing gender attitudes towards health and increasing women’s status continuously in China. Chinese women are playing a bigger role in family decisions and gradually becoming the major contributors to family decisions [32]. In additional, the Chinese government considers women's health as an area of priority in promoting gender equality, and it pays greater attention to meeting women's demands for healthcare service at all periods of their life. Therefore, understanding the health status and healthcare needs of women can help us solve problems in medical care delivery.

Health care seeking behavior will certainly change in the future, because of the increasing aging population, the growing health consciousness of the general public, improvements in the health insurance system, and in the primary care delivery, as well as widespread healthcare information. The results of this study would be useful for highlighting health care system reform, the formulation of better health care policy and improving medical education to meet the expanding demands of the people. More comprehensive researches are needed to study the extent of the gap of healthcare utilization between urban and rural populations, which may help to achieve targeted objectives of health care policy.

## Supporting Information

Table S1 **Definition of terms in the manuscript.**
(DOC)
