# Supplementary material for: The Ecology of Medical Care in Beijing
Source: PLoS One. 2013 Dec 5;8(12):e82446. doi: 10.1371/journal.pone.0082446 (PMC3855438; doi:10.1371/journal.pone.0082446)
Supplement: Table S1 — Definition of terms in the manuscript. (DOC) [file pone.0082446.s001.doc]

Table S1. Definition of terms in the manuscript.

| Definition of terms | |
| --- | --- |
| Term | Definition |
| Final estimate | The number of persons per 1000 in Beijing who had each type of event. |
| People | An urban, 15 age above member of Beijing urban resident population in 2012. |
| Month | 30 days immediately preceding interviews conducted between March and May of 2012. |
| Report symptoms | Any self-reported discomfort, illness or injury. |
| Consider seeking health care | An affirmative response to the question, “In the past 30 days, have you considered or intended to seek medical care for any health problem, although you may not have actually visited a health care professional ” or an affirmative response to at least one of the following:(1) visit to a community health service centre or station, (2) visit to a secondary hospital, (3) visit to a tertiary hospital, (4) accept a traditional Chinese medical therapy, (5) accept a western allopathic medical therapy, (6) visit to an emergency department, (7) inpatient hospital stay. |
| Visit to a primary care | A visit to a general practitioner or family physician in community health service centre or station. |
| Visit to a hospital-based outpatient clinic | A visit to a secondary hospital outpatient department including traditional Chinese medical practitioner and western allopathic medical practitioner work there.  A visit to a tertiary hospital outpatient department including traditional Chinese medical practitioner and western allopathic medical practitioner work there. |
| Accept a traditional Chinese medicine therapy | Diagnosis with four diagnostic processes, which are inspection, listening and smelling, inquiry and palpation. Consume OTC Chinese patent medication (tablets or powder), buy or consume Chinese herbs, or buy Chinese medical cream or liquid for external use, accept acupuncture, massage or moxibustion therapy. |
| Accept a western medicine therapy | Diagnosis with advanced medical equipment (X-ray, computer tomography or endoscopy) and laboratory tests. Consume OTC western medication, or buy medical cream for external use. |
| Visit to an emergency department | A visit to an emergency department of any hospital regardless of using 122 or 999 or not. |
| Inpatient hospital stay | A stay of any duration after admission to a facility licensed or registered as a hospital by a province to provide diagnostic, observed and therapeutic services for a variety of medical conditions, both surgical and non-surgical. |
